# Personality Disorder and Changes in Affect Consciousness: A 3-Year Follow-Up Study of Patients with Avoidant and Borderline Personality Disorder

**DOI:** 10.1371/journal.pone.0145625

**Published:** 2015-12-23

**Authors:** Eivind Normann-Eide, Merete Selsbakk Johansen, Tone Normann-Eide, Jens Egeland, Theresa Wilberg

**Affiliations:** 1 Department of Personality Psychiatry, Division of Mental Health and Addiction, Oslo University Hospital, Oslo, Norway; 2 Research Department, Division of Mental Health and Addiction, Vestfold Hospital Trust, Tonsberg, Norway; 3 Institute of Clinical Medicine, University of Oslo, Oslo, Norway; 4 Department of Research and Development, Division of Mental Health and Addiction, Oslo University Hospital, Oslo, Norway; Central Institute of Mental Health, GERMANY

## Abstract

Personality disorders (PDs) are highly prevalent in patients receiving psychiatric services, and are associated with significant personal and social costs. Over the past two decades, an increasing number of treatment studies have documented the effectiveness of treatment for patients with PDs, especially when it comes to reduction of symptom distress, risk taking behavior, self-harm, or suicide attempts. However, less is known about the more complex aims of improving the personality structure itself, such as identity- and interpersonal disturbances. Emotional dysfunction is closely associated with PD pathology. The present study investigated changes in affect consciousness (AC) in patients with avoidant or borderline PD, and how these changes were associated with clinical status after 3 years of follow-up. The study included 52 individuals; 79 percent were females, and mean age was 30 years. The evaluations included the Affect Consciousness Interview, Symptom Checklist-90-R, Circumplex of Interpersonal Problems, the Index of Self-Esteem, and three domains (Identity Integration, Relational Capacities, and Self-Control) of the Severity Indices of Personality Problems (SIPP-118). There was a significant increase in the Global AC and AC scores for most of the specific affects from baseline to follow-up. As the present study did not include a control group, it cannot be concluded that changes in AC are effects of psychotherapy, and the possibility of age-related maturation processes cannot be excluded. The change in Global AC contributed significantly to explained variance in the follow-up levels of Circumplex of Interpersonal Problems, and the two SIPP-118 domains Relational Capacities and Identity Integration. Improved AC was not associated with change in the Self-Control domain or the Global Severity Index of Symptom Checklist-90-R. The results suggest that AC may be altered for patients with borderline and avoidant PDs, and this is the first study to report that improvement in AC contribute significantly to the variance in the self- and interpersonal domains of personality functioning.

## Introduction

Personality disorders (PDs) are prevalent in the general population and in those receiving psychiatric services, and are associated with significant personal and social costs due to severe symptom distress and impaired psychosocial functioning [1–4]. It was previously thought that patients with PDs gain little benefit from psychotherapy; although, an increasing number of randomized controlled treatment studies during the last two decades have led to a more optimistic view regarding the treatment potential for these patients [5–11]. However, most treatments are aimed at reducing acute symptoms and PD related epiphenomena, such as self-harm, suicide attempts, or risk taking behavior; while, less is known about the more complex aims of improving aspects of the personality structure itself, such as identity disturbances and interpersonal dysfunction [12].

The general definition of a PD in the DSM-5 describes problems in the area of affectivity (i.e., the range, intensity, lability, and appropriateness of emotional responses) and emphasizes this as one of four core PD features, together with deviations in cognition, interpersonal functioning, and impulse control [13]. Accordingly, even if most studies of emotional problems in PDs have focused on borderline personality disorder (BPD), there is increasing empirical evidence that other types of PDs are associated with maladaptive affect regulation as well [14–16], including difficulties in identifying, tolerating, and distinguishing affects, and in communicating affects to others [17–21]. However, it is unclear to what extent such affective difficulties may improve over time in patients with PDs, mainly due to a lack of empirical studies. As it is hypothesized that patients with BPD and avoidant personality disorder (APD) are characterized by different kinds of affective dysfunction [17, 21, 22], they represent a potential variety of emotional difficulties. Moreover, BPD and APD are among the most common PDs in clinical settings [3, 23–25], and thereby of particular interest when studying affects in the context of psychotherapy.

Several psychotherapeutic orientations are directed at the patient’s affective experiences and recognize that overcoming affect avoidance and improving the ability to regulate and adjust emotional responses to current contexts are the main routes to changing the individual’s self-experience and his/her relations to others [26–31]. Yet, with a few notable exceptions, there are few empirical investigations of the relationship between actual change in affective experiences and treatment outcome in patients with PDs.

In a study of patients with BPD who received either Dialectic Behavioral Therapy or General Psychiatric Management, which included psychodynamic individual therapy as one of its elements, McMain and colleges [32] found that the patients, in both treatment conditions, achieved a better balance between positive and negative emotions and an increased ability to identify feelings. The improved ability to experience positive relative to negative affects was associated with reductions in symptom distress and interpersonal problems after one year of treatment. In a sample of patients with cluster C PDs (i.e., avoidant, dependent, and obsessive-compulsive PDs) allocated to short term dynamic therapy or cognitive therapy, decreases in the levels of inhibitory affects (i.e., anxiety, shame, guilt) and increases in levels of previously avoided activating affects (i.e., sadness, anger, closeness) predicted higher self-compassion towards the end of therapy [33]. In the same sample, Berggraf and colleges [34] found that a higher than usual experience of activating affects (i.e., anger, pride, closeness, grief, healthy fear, and sexual needs) in a given therapy session was associated with more realistic and compassionate views of self and others in the next session.

One approach to the study of affects in psychotherapy is represented by the concept of affect consciousness (AC), defined as the individual’s capacity to consciously perceive, tolerate, reflect upon, and express experiences of basic affective activation [28, 35]. Accordingly, the AC concept encompasses several aspects of affect processing and integration that are assumed to be crucial for adequate affect regulation [36]. As emphasized by Choi-Kain and Gunderson [37], AC is related to other “conceptual cousins”, such as mentalized affectivity and alexithymia. However, levels of AC are operationalized and assessed by the semi-structured Affect Consciousness Interview (ACI) [38], which enables an evaluation of AC based on the examination of awareness, tolerance, and non-verbal as well as verbal expression of eleven specific affects. Using this methodology, empirical studies have shown that impaired AC is related to a wide range of psychological and interpersonal disturbances, supporting the validity of the AC construct [35, 39–51].

Improved AC refers to more adaptive implicit and explicit ways of coping with activated affects, and therapeutic change is hypothesized to occur when the basic organizing principles of maladaptive affect processes are changed [52]. However, despite this assumption, only a few studies have investigated the degree to which AC actually changes during treatment. Monsen and colleges [40, 41] found an improvement in AC in a small sample of patients with PDs and psychosis with AC evaluations at the treatment start, treatment termination, and at follow-up 5 years after the end of treatment. Focusing on AC was one of the central elements in the psychotherapy, and the effect size (ES) for the overall change in AC was large (ES = 2.30). The patients also improved with respect to both Axis I and Axis II disorders; however, the authors did not investigate whether such improvements were associated with changes in AC.

In a second treatment study of individuals with chronic pain disorders, AC-oriented psychotherapy was compared with physical therapy and pain-reducing medication [42]. Significant improvement was found in the group receiving AC-oriented treatment, in terms of reduced somatic pain, symptom distress, interpersonal problems, general psychopathology, and especially regarding AC (ES = 2.63). No change in AC was found in the control group. As there were statistically significant differences in outcome between the treatment groups in all clinical variables, the authors argued that the amelioration of psychopathology in the AC treatment group was related to the AC-focused psychotherapy. However, the relationship between the change in AC and change in other clinical variables was not investigated.

The final, and to our knowledge the only, study that has investigated whether improved AC is associated with clinical change was a treatment study of patients with anxiety disorders, with approximately half having co-occurring cluster C PDs [43]. All patients were treated with schema-focused psychotherapy, comprising both group and individual sessions aimed at changing schemas and affective avoidance, as well as behavioral experiments. A significant change in AC between treatment initiation and 1-year follow-up was reported. However, although the results showed that higher levels of AC at baseline predicted a reduction of avoidant personality traits, changes in AC during treatment were not associated with changes in any of the cluster C indexes. As an increased likelihood of having alexithymia problems and low levels of AC are reported in patients with cluster C pathology [17, 21], it might be that the treatment period in this study, just above one year, was too limited for the patients with relatively low levels of AC in particular, to benefit in the same way as the patients with relatively high initial level of AC. Moreover, change in affective function might be related to other factors, such as time, or age-related maturation, and these conditions were not controlled for in the previous studies of change in affective function. Hence, even though there is incipient evidence that AC may change during psychotherapy for patients with PDs, the relationship between change in AC and clinical improvement remains unclear.

In a previous study of the present sample, we found that lower levels of AC were associated with more interpersonal and self-esteem problems when measured cross-sectionally at two points of time, while there was no significant association between AC and general symptom distress [46]. The present study is an extension of our previous study and aimed to analyze changes in AC and its association with clinical status. The sample comprised patients with BPD and APD who participated in a treatment study and, as a group, improved clinically in from baseline evaluation to 3-year follow-up.

Furthermore, it is widely recognized that there is a dimensional continuum between normal and pathological personalities, and that the clinical manifestations of PDs may vary depending on the severity of the personality pathology [53–55]. In the alternative model of PDs in the DSM-5, (section III, for further study), PD severity is defined by varying levels of personality functioning [13]. According to this model, disturbances in the self and relational domains of personality functioning constitute the core of personality psychopathology. To date, surprisingly few empirical studies have investigated associations between changes in affective functioning and such central aspects of personality functioning.

The first aim of the present study was to investigate the extent to which AC changed from the treatment start to the 3-year follow-up in the sample of patients with BPD and APD. Based on our literature review, we hypothesized that there will be significant changes in AC. The second aim was to analyze the associations between changes in AC and clinical status at follow-up, with regard to reduction in symptom distress and improvements in self and relational functioning. We expected that there would be a main effect of pretreatment clinical status. However, we also expected that acquired changes in the way of handling affects would exert a moderate but significant additional effect on clinical improvement, over and above what could be predicted from pretreatment status.

## Methods

### Setting and Design

The present study is part of the Ulleval Personality Project (UPP), a randomized clinical study of long-term treatment for patients with PDs, conducted at the Department for Personality Psychiatry, Oslo University Hospital [56–60]. The overall aim of the UPP was to investigate the effect of a step-down day hospital treatment program compared with outpatient individual psychotherapy. All participants were randomly allocated to one of the two treatment conditions. The participants were referred from outpatient clinics, psychotherapists in private practice, or general practitioners. Exclusion criteria were schizotypal PD, antisocial PD, ongoing drug or alcohol dependence, psychotic disorder, bipolar I disorder, untreated ADHD (adult type), pervasive developmental disorder (e.g., Asperger’s syndrome), organically contingent symptoms, and homelessness. The participants went through an extensive evaluation at baseline, with repeated assessments after 8 months, 18 months, 3 years, and 6 years. In the subsample of patients with either BPD or APD, AC was assessed at baseline and at the 3-year follow-up. Written informed consent was obtained from participants after complete description of the study. The study was approved by the Norwegian State Data Inspectorate and the Regional Committee for Medical Research Ethics.

### Assessments

#### Affect Consciousness

The AC was assessed using the ACI, a one and a half to two hours semi-structured interview, developed to assess the consciousness and integration of 11 basic affects: (1) Interest/Excitement, (2) Enjoyment/Joy, (3) Fear/Panic, (4) Anger/Rage, (5) Disdain/Contempt, (6) Shame/Humiliation, (7) Sadness/Despair, (8) Envy/Jealousy, (9) Guilt/Remorse, (10) Disgust/Revulsion, and (11) Tenderness/Care. Each affect was assessed using a 9-point Affect Consciousness Scale (1 = low, 9 = high) regarding the following aspects: Awareness, Tolerance, Emotional (nonverbal) expression, and Conceptual (verbal) expression [38]. To examine each aspect, the interviewer asked about the following for each affect: (i) social or interpersonal scenes in which the affect is activated; (ii) how the patient becomes aware of and recognizes the affect in terms of physical and mental sensations/thoughts (Awareness); (iii) how the affect impacts upon the patient, how the patient copes with the affect, and to what extent the patient utilizes the signal function of affects (Tolerance); (iv) to what extent and how the affect is expressed nonverbally (Emotional expression); and finally, (v) to what extent and how the affect is expressed verbally (Conceptual expression). From these ratings, one can differentiate scores on three levels of specificity:

- The mean score of all aspect scores across all affects (i.e., Global AC).
- The mean score of each aspect measured across all affects (i.e., Awareness, Tolerance, Emotional expression, and Conceptual expression).
- The mean score of each affect category, measured across the four aspects (e.g., Interest/Excitement or Anger/Rage).

All interviews were videotaped and scored according to the manual for the ACI [38]. Three experienced psychotherapists specifically trained in the ACI conducted the interviews at baseline and at the 3-year follow-up. Two of the three interviewers performed the ratings (neither rated their own interviews). Both raters were blinded to each patient’s background, diagnosis, and treatment condition. Twenty interviews were rated by a third, external, independent rater. Ten interviews were randomly selected from baseline and follow-up. The reliability coefficients (ICC 2.1) were 0.74 for the Global AC index, and 0.53, 0.65, 0.73, and 0.88 for Awareness, Tolerance, Emotional expression, and Conceptual expression, respectively. The reliability was satisfactory for nine of the eleven specific affects, ranging from 0.67 for Disdain/Contempt to 0.87 for Tenderness/Devotion. However, the reliability was unsatisfactory for Sadness/Despair (0.54) and Envy/Jealousy (0.55).

#### Axis I and axis II diagnoses

Axis I diagnoses were based on the Mini International Neuropsychiatric Interview [61]. Axis II diagnoses were determined according to the 4^th^ edition of the Diagnostic and Statistical Manual of Mental Disorders and the Structured Clinical Interview for DSM-IV Axis II Disorders (SCID-II) [62]. Experienced clinicians performed the interviews at the treatment start. An external independent rater evaluated 24 videotaped SCID-II interviews. Kappa values were 0.75 for APD and 0.66 for BPD, indicating acceptable diagnostic reliability.

#### Symptom distress

The Symptom Checklist-90-Revised (SCL-90-R) [63] was used to measure symptom distress. The SCL-90-R is a self-reported questionnaire with a 0 to 4 Likert scale. It is designed to cover the major symptoms of psychological distress, represented in nine dimensions that can be meaningfully summarized in a Global Severity Index (GSI). A higher score indicates more symptom distress.

#### Interpersonal problems

The Circumplex of Interpersonal Problems (CIP) [64] was used to assess interpersonal personal problems. This self-reported questionnaire is a 48-item version of the Inventory of Interpersonal Problems [65]. It comprises eight subscales that are summarized in a CIP index. The items are rated on a five-point Likert scale from 0 to 4; a higher score indicates more interpersonal problems.

#### Self-Esteem

Self-esteem was assessed using the Index of Self-Esteem (ISE) [66, 67]. The ISE is a 25-item self-evaluative questionnaire that measures the degree or severity of a respondent’s self-esteem problems. The scale is scored from 0 to 100; a score of 0 indicates that the respondent has none of the attributes and 100 represents the highest possible distress level. Respondents that score >30 are assumed to have clinically significant self-esteem problems. Cronbach’s alpha of the Norwegian ISE scale in the UPP sample was 0.91, which indicated good internal consistency [23].

#### Personality functioning

Severity Indices of Personality Problems (SIPP-118) is a self-report questionnaire for the assessment of core components of (mal)adaptive personality functioning that are believed to be changeable [68–70]. The instrument covers many of the personality domains conceptualized as core aspects of the personality pathology in the alternative model in the DSM-5 [13, 69]. The SIPP-118 consists of 118 4-point Likert scale items covering 16 facets of personality functioning that cluster in five higher-order domains [68]. Higher scores indicate more adaptive functioning. The SIPP-118 questionnaire was translated from English to Norwegian by our research group and then translated back to English by an independent bilingual translator. Although the factor structure of SIPP-118 has not been fully settled [69], three studies have reported good psychometric properties in adults and adolescents [68, 71], including cross-national consistency in adult PD populations [72]. In the present study we utilized three of the five personality functioning domains in the SIPP-118, i.e., Identity Integration, Relational Capacities, and Self-Control, based on the assumption that changes in AC would be particularly relevant to these areas of functioning [70].

### Participants

Eighty-one patients with BPD or APD were included in the UPP study. For four of these patients, the ACI was not feasible due to technical failure of the video recordings on two occasions, and the inability to conduct the interview on two other occasions. Three other patients were initially included, but were excluded after initial treatment; one received a diagnosis of Asperger’s disorder, another had prefrontal organic brain damage, and the third was judged to be on the border of mental retardation. Of the remaining 74 patients, 52 (70%) attended the 3-year follow-up examination and were included in the present analyses. However, one more patient was excluded in the analyses of the SIPP-118 domains. This patient’s scores on SIPP-118 were judged as invalid due to the measurement of severe personality functioning in several other clinical assessments combined with extremely high values on the SIPP domains.

The socio-demographic and clinical characteristics at baseline are presented in Table 1. Sixty-five percent of the patients had BPD and 50% had APD. Thirty-seven percent of the patients had two or more PD diagnoses and the distribution of co-occurring PD diagnoses was: Paranoid PD 12%, Obsessive Compulsive PD 12%, Dependent PD 6%, Narcissistic PD 2%, and Schizoid PD 2%. The mean number of PD traits was 16 (SD = 6.2). Of the included patients, 28 (54%) were randomly allocated to the Step-down condition and 24 (46%) to the Outpatient condition. There were no statistically significant differences between the two treatment conditions in socio-demographic or clinical characteristics at baseline, except for a higher rate of patients with self-harm behaviors over the last year in the Step-down condition (48%) compared with the outpatient condition (17%) (P = .021). The patients who did not attend the 3-year follow-up (Step-down 33%, Outpatient 26%, ns) had a significantly higher GSI (P = .009) at baseline, had a higher number of Axis I disorders (P = .001), and were more frequently diagnosed with substance abuse (P = .008), alcohol abuse (P = .006), and panic disorder (P = .042) compared with those included.

**Table 1:** Socio-demographic and Clinical Characteristics at Baseline.

	Total Sample (n = 52)
Age, mean (SD)	30 (7)
Females, %	79
Married/Cohabiting, %	16
Completed high-school, %	46
Unemployed, %	39
Attempted suicide last year, %	12
Self-harming behavior last year, %	33
No. PD criteria, mean (SD)	16 (6.2)
Personality disorder (PD) diagnosis	
Borderline PD, %	65
Avoidant PD, %	50
Paranoid PD, %	12
Obsessive-compulsive PD, %	12
Dependent PD, %	6
Narcissistic PD, %	2
Schizoid PD, %	2
No. symptom disorders, mean (SD)	3.2 (1.4)
Mood disorders, %	83
Anxiety disorders, %	85
Substance abuse, %	23
Eating disorders, %	12

### Treatments and treatment received

#### Step-down treatment

The patients allocated to Step-down treatment first attended an 18-week day-hospital treatment that utilized a combination of psychodynamic and cognitive-behavioral group therapies 3 to 4 days a week. Most therapists were trained and experienced group psychotherapists [57]. After the initial day-hospital treatment, the patients continued with outpatient combined psychotherapy consisting of weekly group therapy (1.5 hours) for a maximum of 4 years combined with weekly individual therapy for a maximum of 2.5 years.

The individual therapists, mostly located outside the hospital setting, were invited to participate in the study and to select whether they preferred to be involved in the Step-down treatment or the Outpatient treatment condition. For the therapists in the Step-down treatment condition, written treatment guidelines adhered to relational psychotherapy, with references to group analysis, self-psychology, and mentalization, but they did not serve as a standard for treatment adherence. However, clinical and theoretical one-day seminars, including case presentations, were organized twice a year for all therapists. Eleven percent of the patients dropped out of the Step-down treatment before starting outpatient combined therapy. Of the remaining patients, 44% were still attending weekly group therapy at the 3-year follow-up and 32% were still in individual therapy. The median number of group therapy sessions in the outpatient combined treatment at the 3-year follow-up was 52 (range, 7–110). The median number of individual therapy sessions was 45 (range, 5–117).

#### Outpatient treatment

The patients allocated to Outpatient treatment attended open-ended individual psychotherapy that was conducted mainly by specialists in private practice. The therapists were instructed to treat patients according to their own preferred method and practice, and the researchers provided no instructions to the therapists regarding the duration or intensity of psychotherapy, nor did they interfere with any treatment decisions. Thirty-eight percent of the patients in Outpatient treatment were still in individual therapy at the 3-year follow-up. The median number of individual psychotherapy sessions during the 3-year period was 46 (range, 5–258). More detailed descriptions of the two treatment conditions and therapists have been reported elsewhere [57–59].

### Statistical Analysis

All the analyses were performed using SPSS statistics version 18. We used independent sample t-tests to evaluate statistical relationships between continuous and categorical variables, chi-square statistics to test associations between categorical variables, and Pearson’s correlation coefficients for the association between continuous variables. The rank order stability of global AC from baseline to follow-up was assessed by Spearman’s rho; whereas, changes in Global AC and the 11 specific affects were examined using paired sample t-tests. Within-group pre-post effect sizes (ES) were computed using Cohen’s *d* with baseline SDs as standardization. According to Cohen [73], the ES may be characterized as *small* (0.2–0.5), *medium* (0.5–0.8), or *large* (>0.8).

To investigate the relationship between change in Global AC and clinical status at follow-up, we computed an AC change score and ran multiple linear regression analyses with GSI, CIP, ISE, and SIPP-118 domains at the 3-year follow-up as dependent variables. At baseline, AC was positively correlated with age (r = 0.30, P = .034) and the female participants had significantly higher AC scores than the males (AC = 3.66, SD = 0.45 versus AC = 3.29, SD = 0.59; P = .026). Therefore, gender and age were entered as independent variables, followed by the baseline level of the dependent variable, and the baseline level of Global AC. The AC change score was entered as the last independent variable to examine whether an altered AC contributed to explained variance at 3-years follow-up beyond the control variables. Finally, as the reliability was unsatisfactory for the AC aspect of Awareness and the affects Sadness/Despair and Envy/Jealousy we computed a new Global AC score, excluding these variables. The regression analyses were then repeated with the new Global AC variable. There were small variations in n across analyses owing to some missing data.

## Results

The mean level of Global AC changed from 3.58 (SD = 0.50) at baseline to 4.00 (SD = 0.58) at the 3-year follow-up. This change was statistically significant (P < .001) (Table 2). There were statistically significant increases in AC for eight of the eleven specific affects: Interest/Excitement (P = .001), Enjoyment/Joy (P < .001), Fear/Panic (P < .001), Anger/Rage (P < .001), Shame/Humiliation (P = .002), Sadness/Despair (P = .010), Guilt/Remorse (P = .006), and Tenderness/Devotion (P = .004). The ES was in the large range for the changes in Global AC (*d* = 0.84) and the affects Fear/Panic (*d* = 1.05) and Anger/Rage (*d* = 0.77), while the ES was in the medium range for Interest/Excitement (*d* = 0.47), Enjoyment/Joy (*d* = 0.57), Shame/Humiliation (*d* = 0.61), Sadness/Despair (*d* = 0.46), and Guilt/Remorse (*d* = 0.48). There were statistically significant increases in all aspects of AC; Awareness (P = .028), Tolerance (P < .001), Emotional expressivity (P < .001), and Conceptual expressivity (P < .001). The magnitude of the changes was largest for Tolerance (*d* = 0.87) and Conceptual expressivity (*d* = 0.92), and smallest for Awareness (*d* = 0.37). The correlation (rho) between the Global AC at baseline and follow-up was 0.59 (P < .001), indicating moderate rank order stability.

**Table 2:** Mean Levels of AC Scales at Baseline and the 3-Year Follow-Up.

	AC scores: change between baseline and follow-up				
	Baseline Mean (SD)	Follow-up Mean (SD)	*t-value*	*P*	Effect Size
Global AC	3.58 (0.50)	4.00 (0.58)	-6.33	< .001	0.84
Specific affects					
Interest/Excitement	4.28 (1.08)	4.79 (1.11)	-3.39	.001	0.47
Enjoyment/Joy	4.33 (1.18)	5.00 (0.81)	-4.85	< .001	0.57
Fear/Panic	2.87 (0.64)	3.54 (0.72)	-5.96	< .001	1.05
Anger/Rage	3.14 (0.73)	3.70 (0.91)	-3.87	< .001	0.77
Disdain/Contempt	3.50 (1.06)	3.61 (1.33)	-0.61	.544	0.10
Disgust/Revulsion	3.92 (1.17)	4.03 (1.35)	-0.69	.469	0.09
Shame/Humiliation	2.99 (0.87)	3.52 (0.88)	-3.26	.002	0.61
Sadness/Despair	3.12 (0.84)	3.51 (0.90)	-2.69	.010	0.46
Envy/Jealousy	3.18 (0.91)	3.31 (0.81)	-0.92	.361	0.14
Guilt/Remorse	3.77 (0.86)	4.18 (0.93)	-2.88	.006	0.48
Tenderness/Care	4.32 (1.21)	4.74 (1.03)	-2.99	.004	0.35
Integrating aspects					
Awareness	4.59 (0.56)	4.80 (0.67)	-2.26	.028	0.37
Tolerance	3.51 (0.49)	3.94 (0.61)	-5.98	< .001	0.87
Emotional expressivity	3.16 (0.67)	3.60 (0.69)	-5.30	< .001	0.65
Conceptual expressivity	3.04 (0.66)	3.65 (0.70)	-6.55	< .001	0.92

As all participants in the sample either had BPD, APD or both PD diagnoses, we investigated whether the PD groups differed regarding change in Global AC. We first compared patients who had either BPD (n = 26) or APD (n = 18). The change in AC did not differ significantly between the groups (P = 0.173). Then, we included the patients with both diagnoses, first, in the BPD group, and then in the APD group. Neither of the between-group differences were significant (P = 0.180 and P = 0.296, respectively).

Taking into account the potential effect of age, a linear regression analysis was conducted. Change in Global AC score was entered as the dependent variable, whereas age and Global AC at baseline were entered as independent variables. Age did not contribute statistical significantly to the explained variance of change in Global AC (P = 0.466).

At baseline, 72% of the patients were treated with medications, mostly antidepressants. From baseline to follow-up there was a reduction in use of antidepressants from 53% to 33% (P<0.001). However, change in use of antidepressants was not associated with change in Global AC (P = 0.200).

The sample improved in both symptoms and personality functioning during the follow-up period, as indicated by significant decreases in levels of GSI (from 1.68 (SD = 0.64) to 1.17 (SD = 0.71); P < .001; ES = 0.80), CIP (from 1.71 (SD = 0.50) to 1.42 (SD = 0.55); P = .001; ES = 0.58), and ISE (from 58.69 (SD = 11.07) to 50.29 (SD = 13.89); P < .001; ES = 0.76), as well as increases in the SIPP-118 domains Identity Integration (from 2.04 (SD = 0.51) to 2.77 (SD = 0.73); P < .001; ES = 1.43), Relation Capacities (from 2.35 (SD = 0.64) to 2.80 (SD = 0.70); P < .001; ES = 0.70), and Self-Control (from 2.44 (SD = 0.62) to 2.98 (SD = 0.60); P < .001; ES = 0.87).

To investigate whether change in the Global AC score was associated with clinical status, the 3-year follow-up scores on the GSI, CIP, ISE, and the SIPP-118 domains—Identity Integration, Relational Capacities, and Self-Control were entered as dependent variables in six separate linear regression analyses. The independent variables were entered in the following order: gender, age, baseline scores for the respective clinical measure, baseline Global AC, and finally AC change. As shown in Table 3, for all clinical measures, the respective baseline levels explained a significant part of the variance at the 3-year follow-up (GSI: 25.9%, P < .001; CIP: 19.5%, P = .002; ISE: 28.3%, P < .001; Identity Integration: 8.5%, P = .047, Relational Capacities: 38.5%, P < .001; Self-Control: 27.5%, P < .001). Gender and age were not associated with the follow-up scores on the GSI, CIP, or ISE. After controlling for the previous variables, the AC change explained an additional 7.7% of the variance in CIP (P = .033), 8.6% of the variance in Identity Integration (P = .026), and 8.7% of the variance in Relational Capacities (P = .005) at follow-up. The AC change was not significantly related to follow-up scores on the GSI or Self-Control levels. The contribution of the AC change was close to significant (P = .076) for the variance in ISE at follow-up.

**Table 3:** Multiple Regression Analyses Using the GSI, CIP, ISE, and SIPP-118 Domains (Identity Integration, Relational Capacities, and Self-control domain) at the 3-Year Follow-Up as Dependent Variables.

		ΔR^2^	ΔF	*P*
**GSI**	Gender	.019	.906	.346
	Age	.021	.945	.326
	GSI baseline	.259	6.279	< .001
	Global AC baseline	.021	5.075	.256
	Global AC change	.009	4.138	.447
	*R* ^*2*^ adjusted	25.0%		
**CIP**	Gender	.006	.293	.591
	Age	.008	.328	.547
	CIP baseline	.195	3.875	.002
	Global AC baseline	.051	3.778	.092
	Global AC change	.077	4.267	.033
	*R* ^*2*^ adjusted	25.8%		
**ISE**	Gender	.002	.070	.792
	Age	.037	.857	.207
	ISE baseline	.283	6.636	< .001
	Global AC baseline	.027	5.492	.197
	Global AC change	.050	5.307	.076
	*R* ^*2*^ adjusted	32.4%		
**SIPP-118:**	Gender	.002	.090	.763
Identity	Age	.129	3.167	.017
Integration	Identity Integration baseline	.081	3.676	.047
	Global AC baseline	.079	4.105	.041
	Global AC change	.086	4.721	.026
	*R* ^*2*^ adjusted	29.7%		
Relational	Gender	.017	.763	.387
Capacities	Age	.084	3.924	.054
	Relational Capacities baseline	.385	12.943	< .001
	Global AC baseline	.034	10.852	.100
	Global AC change	.087	12.085	.005
	*R* ^*2*^ adjusted	55.7%		
Self-	Gender	.034	1.536	.222
Control	Age	.075	2.591	.066
	Self-Control baseline	.275	8.550	< .001
	Global AC baseline	.000	6.261	.909
	Global AC change	.015	5.204	.327
	*R* ^*2*^ adjusted	32.4%		

To account for any possible influence of insufficient reliability of some aspects of the AC measure, we repeated the analyses with the revised Global AC. The significance levels of the former analyses were confirmed and strengthened in most respects, especially regarding the ISE. Under this condition, the contribution of the AC change to the ISE at the 3-year follow-up was significant and explained an additional 7.2% of the variance (P = .030).

## Discussion

We found support for the first hypothesis that the participants AC would improve from baseline to follow-up, 3-years after treatment start. We interpret this as evidence that it is possible to change from non-adaptive to more adaptive ways of utilizing one’s affects, which is one aspect of what constitute personality disorders. Such a change may be valuable by itself, but the main question remains: Is change in AC associated with clinical improvement or less interpersonal conflicts? The second expectation, that an altered AC would also be related to positive changes in clinical status, i.e., severity of interpersonal and self-related problems, and symptom distress, was partly supported. In this section, we will discuss the two main findings in more detail, and compare the results with previous comparable research.

The finding that the Global AC score improved from baseline to the 3-year follow-up concurs with previous psychotherapy studies reporting improved AC in samples of patients with PDs, anxiety disorders and chronic pain disorders, measured after treatment- and follow-up periods, ranging from 1 to 7 years [40–43]. Furthermore, there were improvements in AC for most affects; for example, the ESs were large for Fear/Panic and Anger/Rage, and ranged from low to moderate for the other affects. The increase in AC did not reach the level of statistical significance for Disdain/Contempt, Disgust/Revulsion, or Envy/Jealousy, suggesting that changes in these particular affects are more difficult than for others.

However, it might be that particular affects, such as fear and anger, are more regularly addressed in psychotherapy, while envy, disgust, or contempt are experienced as more chaotic, elusive, or associated with shame, and may need special attention and intervention, or a particularly trustful therapeutic relationship to be brought forward and become a subject for exploration in the therapeutic dialogue. Influenced by Silvan Tomkins’ script theory, Magai [74] suggests that shame, if repeatedly socialized and associated with other affects, creates affect-shame binds. As a result, when the affect associated with shame is activated, shame is experienced and the initial affect is inhibited. In the present study, the absence of change in AC for particular affects could be due to such affect-shame binds, inhibiting the exposure of these affects in psychotherapy.

Despite the significant changes in AC, the ESs did not reach the same levels as in previous studies of AC [40–43]. This discrepancy might be related to the different versions of the AC scale and scoring procedures used in the former and present study, making comparisons between studies somewhat difficult. In the present and most recent version of the ACI, eleven affects are scored on a 9-point scale; while, in the previous studies nine affects were scored on a 5-point scale. There is a need for more knowledge of the psychometric properties of the last version of the AC scales, their sensitivity to change, and what might be expected as a normal AC level.

On the other hand, the differences in ESs could be influenced by patient characteristics. It might be that AC problems are more chronic, or resistant to change in certain disorders or types of PDs. The present sample comprised patients with BPD and APD, which are assumed to be characterized with affect dysregulation and affect avoidance respectively [21, 27, 75, 76]. Interestingly, in this sample of poorly functioning patients, we did not find any difference in AC change between the PD groups.

Finally, differences in ESs between the studies could also be due to treatment features. In two of the former studies evaluating changes in AC [40–42], the patients received psychotherapy especially developed to enhance AC. Working with affects, both within the therapeutic relationship and in other significant relationships in the patient’s life, is central to many forms of contemporary psychotherapies, across different theoretical perspectives. Yet, common clinical sense would suggest that AC focused psychotherapy, which is developed within the context of the AC methodology (i.e., in which the therapist consistently focuses on helping the patient to recognize and label individual affects, and subsequently facilitates reflection and a more effective communication of affects) enhances the potential for change in AC. Regarding the present study, however, the results must be interpreted with caution as it did not include a no-treatment clinical control group. Even though the present change in AC was not related to age, we can not exclude the possible effects of age-related maturation, and more studies are needed regarding this issue.

The second main finding of the present study was how clinical status at follow-up were related to the improvement in AC. Cross-sectional studies on AC have reported an association between the level of AC and severity of the psychopathology, i.e., self-esteem and interpersonal problems [35, 44–46]. The present results complement these studies, and to our knowledge this is the first study to report that improved AC is related to follow-up measures of self-relatedness and interpersonal function, which are some of the core markers of maladaptive personality functioning.

More specifically, improved AC was associated with the Identity Integration domain of the SIPP-118, and this explained almost 9% of the variance at follow-up, whereas the relationship with self-esteem problems was significant in the control analysis, i.e., after removing the affects and aspect with insufficient reliability. Identity Integration, as defined by the SIPP-118, refers to the ability to tolerate frustration, enjoyment, and to see one’s life as stable, integrated, and purposeful [68, 70]. Furthermore, a relationship between low scores on the Identity Integration domain and the presence of PD has been found in studies of both adults and adolescents [68, 71, 77]. Within the alternative model of the DSM-5 (section III) [13], identity constitutes one of two main elements of self-functioning, and the capability of experiencing, tolerating, and regulating a full range of emotions is defined as an aspect of mature, well-integrated identity. So far, however, few empirical studies have investigated the relationship between emotional functioning and identity. The present results support the notion in section III that adaptive ways of utilizing one’s affects is significant for an individual’s experience of a coherent and purposeful self.

However, the relationship between emotional processing and identity is probably complex. For instance, the sense of identity in subjects with low AC may be depleted by reduced awareness of various affects and their inherent motivational aspects. On the other hand, subjects with low AC may occasionally be so overwhelmed by their affects that their sense of identity is temporarily lost. Nevertheless, increased levels of Identity Integration have been reported in patients with PDs who have received psychotherapy [68, 77–79], and the present results suggest that improvements in AC may contribute to such strengthening of identity.

In regards to interpersonal function, improved AC explained a significant part of the variance at follow-up, when measured with the CIP and the SIPP-118 domain Relational Capacities, which evaluates the capacity for intimacy, enduring relationships, and the ability to feel recognized by others [68, 70]. These results strengthen the evidence from previous clinical studies of the relationships between changes in emotional awareness, balance, and expression and improved interpersonal function in patients with BPD [32] and patients with cluster C PDs [34]. The present results also concur with findings that communication and the sharing of emotions are associated with intimacy and strengthening of social bonds [80–82].

According to Bastiaansen and colleges [69], the SIPP-118 Identity Integration domain captures most of the self-components in the DSM-5, section III; Identity and Self-Direction, whereas, the SIPP-118 Relational Capacities domain covers the relational components; Intimacy and Empathy. Thus, these preliminary findings suggest that improved AC is associated with follow-up measures for the two core domains of personality psychopathology. Accordingly, psychotherapies with patients with PDs that integrate an affective focus and succeed in increasing emotional awareness and recognition, as well as an ability to reflect upon and express a wide range of affects, might help the patients to develop more adaptive personality functioning. However, there is a need for further examination of the potential links between psychotherapeutic processes and emotional and personality growth. Future studies should investigate to what extent, and how these processes are related in various treatments and patient populations.

The lack of relationship between a change in AC and follow-up level of the SIPP-118 Self-Control domain was surprising. This domain includes emotion regulation, effortful control, and aggression regulation [68]. Based on theoretical expectation and clinical intuition, it was expected that improved AC would be related to follow-up levels of this domain as well. There may be several explanations for this finding. First, it has been suggested that patients with APD are characterized with affect phobia or a general suppression of affects [17, 83], while patients with BPD are less able to suppress, or regulate, unpleasant experiences of affects [75, 84, 85]. Thus, the two PD groups in the present sample might represent extremes with respect to either low or high self-control, and neutralize the relationship between AC change and Self-Control. Furthermore, the Global AC summarizes different aspects of emotional functioning and the SIPP-118 domains are also multi-faceted. The present sample was too small to explore whether changes in the different aspects of AC were related to Self-control or certain facets of Self-control. Also, the psychometric properties of the SIPP-118 domains have not been confirmed; for instance, the primary factor loadings of the Aggression Regulation facet on the Self-control domain have differed between samples [68–71]. However, as indicated by our result, there is a possibility that there is no strong relationship between enhanced AC and improved self-control. More studies are needed to clarify these issues.

Theoretically, AC is assumed to be associated with symptom distress [28], and some clinical studies have supported this [35, 44, 45, 51]. Similarly, McMain and colleges [32] reported that an increase in positive versus negative affects was associated with improvements in both general symptom distress and interpersonal function in a sample of patients with BPD. However, in the present study, a change in AC was not, under any conditions, associated with the follow-up level of symptomatic distress, and this result might be related to sample selection. In more narrow psychiatric samples, such as patients with eating disorders [49] or in our previous study based on the present sample [46], AC was not related to symptom distress. Hence, even though patients with more severe PD pathology experience high levels of psychological symptoms overall [86], the clinical variation in a PD sample might be too limited to illuminate a relationship between changes in the level of AC and symptom distress.

The present results should be interpreted in light of some limitations. First, as there was no untreated control group included in the present study, we do not know whether the changes in AC or clinical measures could be attributed to psychotherapy effects, or whether the improvements were due to alterations over time, e.g. age-related maturation. Second, study inclusion was restricted to patients with APD or BPD as their main PD diagnosis. Even though the participants had other co-occurring PD diagnoses, the results may not be generalized to other PD populations or to less disturbed patients. Third, the small sample size increases the possibility of type 2 error. Accordingly, the present results need to be replicated in larger studies and samples that comprise mixed clinical populations and different PDs. Fourth, the present results may be influenced by attrition bias, as the group of patients that did not attend the 3-year follow-up investigation had more severe symptoms and more frequent substance use disorders. Finally, the reliability was not satisfactory for three variables, and the control analyses suggested a stronger relationship between changes in AC and self-esteem in particular.

In conclusion, the present results suggest that patients with PDs have the potential for improvements in AC, and this is the first study to report that such improvement is related to clinical status at follow-up, particularly within the self and interpersonal domains of personality functioning. Thus, psychotherapies that succeed in increasing the patients’ level of affect integration might have the potential to help patients with PDs to develop more adaptive personality functioning. Future studies should be conducted in controlled settings, aimed at expanding our knowledge of how various forms of psychotherapy and treatment programs can facilitate the awareness, tolerance, and communication of affective experiences, as well as investigate the assumed mediating effect of AC on psychotherapy outcome. Although AC is relevant for a broad range of clinical disorders and populations, it may be especially important for the treatment of patients with PDs, for whom emotional dysfunction is a key characteristic.

## Abbreviations

AC: affect consciousness
ACI: Affect Consciousness Interview
PD: Personality Disorder
APD: avoidant personality disorder
BPD: borderline personality disorder
CIP: Circumplex of Interpersonal Problems
SCL-90-R: Symptom Checklist-90-R
GSI: Global Severity Index
ISE: Index of Self-Esteem
SIPP-118: Severity Indices for Personality Problems
ES: effect size
UPP: Ulleval Personality Project

## References

[1] Johnson JG, Cohen P, Kasen S, Skodol AE, Oldham JM. Cumulative prevalence of personality disorders between adolescence and adulthood. Acta Psychiatr Scand. 2008;118: 410–413. 10.1111/j.1600-0447.2008.01231.x 18644003

[2] Torgersen S. The nature (and nurture) of personality disorders. Scand J Psychol. 2009;50: 624–632. 10.1111/j.1467-9450.2009.00788.x 19930262

[3] Zimmerman M, Rothschild L, Chelminski I. The prevalence of DSM-IV personality disorders in psychiatric outpatients. Am J Psychiatry. 2005;162: 1911–1918. 16199838 10.1176/appi.ajp.162.10.1911

[4] Skodol AE; Pagano ME; Bender DS; Shea MT; Gunderson JG; Yen S, et al Stability of functional impairment in patients with schizotypal, borderline, avoidant, or obsessive-compulsive personality disorder over two years. Psychol Med. 2005; 35: 443–451. 15841879 10.1017/s003329170400354x PMC3272760

[5] Svartberg M, Stiles TC, Seltzer MH. Randomized controlled trail of the effectiveness of short-term dynamic psychotherapy and cognitive therapy for cluster C personality disorders. Am J Psychiatry. 2004;161: 810–817. 15121645 10.1176/appi.ajp.161.5.810

[6] Giesen-Bloo J, van Dyck R, Spinhoven P, van Tilburg W, Dirksen C, can Asselt T, et al Outpatient psychotherapy for borderline personality disorder: randomized trail of schema-focused therapy vs transference-focused therapy. Arch Gen Psychiatry. 2006;63: 649–658. 16754838 10.1001/archpsyc.63.6.649

[7] Kernberg OF, Yeomans FE, Clarkin JF. Levy KN. Transference focused psychotherapy: Overview and update. Int J Psychoanal. 2008;89: 601–620. 10.1111/j.1745-8315.2008.00046.x 18558958

[8] Bateman A, Fonagy P. 8-year follow-up of patients treated for borderline personality disorder: mentalization-based treatmet versus treatment as usual. Am J Psychiatry. 2008;165: 631–638. 10.1176/appi.ajp.2007.07040636 18347003

[9] McMain SF, Links PS, Gnam WH, Guimond T, Cardish R, Korman L, et al A randomized trial of dialectical behavior therapy versus general psychiatric management for borderline personality disorder. Am J Psychiatry. 2009;166: 1365–1374. 10.1176/appi.ajp.2009.09010039 19755574

[10] Sempertegui GA, Karreman A, Arntz A, Bekker MH. 2013. Schema therapy for borderline personality disorder: a comprehensive review of its empirical foundations, effectiveness and implementation possibilities. Clin Psychol Rev. 2013;33: 426–447. 10.1016/j.cpr.2012.11.006 23422036

[11] Dimaggio G, Nicolò G, Semerari A, Carcione A. Investigating the personality disorder psychotherapy process. Psychother Res. 2013;23: 625–632. 10.1080/10503307.2013.845921 24252091

[12] Bateman A, Gunderson J, Mulder R. Treatment of personality disorder. Lancet. 2015;385: 735–743. 10.1016/S0140-6736(14)61394-5 25706219

[13] American Psychiatric Association. Diagnostic and statistical manual of mental disorders (5th Ed.). Washington, DC; 2013.

[14] Gross JJ. The emerging field of emotion regulation: An integrative review. Rev Gen Psychol. 1998;2: 271–299.

[15] Joyce AS, Fujiwara E, Cristall M, Ruddy C, Ogrodniczuk JS. Clinical correlates of alexithymia among patients with personality disorder. Psychother Res 2013;23: 690–704. 10.1080/10503307.2013.803628 23731378

[16] Pedersen G, Johansen MS, Wilberg T & Karterud S. Testing Different Versions of the Affective Neuroscience Personality Scales in a Clinical Sample. PLOS ONE. 2014;9: e109394 10.1371/journal.pone.0109394 25289939 PMC4188588

[17] Nicolò G, Semerari A, Lysaker PH, Dimaggio G, Conti L, D’Angerio S, et al Alexithymia in personality disorders: correlations with symptoms and interpersonal functioning. Psychiatry Res 2011;190: 37–42. 10.1016/j.psychres.2010.07.046 20800288

[18] Lecours S, Bouchard MA. Verbal elaboration of distinct affect categories and BPD symptoms. Psychol Psychother T. 2011;84: 26–41. 10.1111/j.2044-8341.2010.02006.x 22903829

[19] Berkin M, Wupperman P. Emotion regulation and mental health. Curr Opin Psychiatry. 2012;25: 128–134. 10.1097/YCO.0b013e3283503669 22262030

[20] Ridings LE, Lutz-Zois CJ. Emotional dysregulation and Borderline Personality Disorder: Explaining the link between secondary psychopathy and alexithymia. Pers Individ Dif. 2014;57: 14–19. 10.1016/j.paid.2013.09.008

[21] Johansen MS, Normann-Eide E, Normann-Eide T, Wilberg T. Emotional dysfunction in avoidant compared to borderline personality disorder: A study of affect consciousness. Scand J Psychol. 2013;54: 515–521. 10.1111/sjop.12076 24107113

[22] Koenigsberg HW, Denny BT, Fan J, Liu X, Guerreri S, Mayson SJ, et al The Neural Correlates of Anomalous Habituation to Negative Emotional Pictures in Borderline and Avoidant Personality Disorder Patients. Am J Psychiatry. 2014;171: 82–90. 10.1176/appi.ajp.2013.13070852 24275960 PMC3947284

[23] Lynum LI, Wilberg T, Karterud S. Self-esteem in patients with borderline and avoidant personality disorders. Scand J Psychol. 2008;49: 469–477. 10.1111/j.1467-9450.2008.00655.x 18564322

[24] Hummelen B, Wilberg T, Pedersen, Karterud S. An investigation of the validity of the *Diagnostic and Statistical Manual of Mental Disorders*, *Forth Edition* avoidant personality disorder construct as a prototype category and the psychometric properties of the diagnostic criteria. Compr Psychiatry. 2006; 47:376–383. 16905400 10.1016/j.comppsych.2006.01.006

[25] Chiesa M, Bateman A, Wilberg T, Friis S. Patients’ characteristics, outcome and cost-benefit of hospital-based treatment for patients with personality disorder: A comparison of three different programmes. Psychol Psychother-T. 2002; 75:381–392 10.1348/147608302321151899 12674121

[26] McCullough Vaillant L. Changing Character: Short-Term Anxiety-Regulating Psychotherapy for Restructuring Defenses, Affects, and Attachment. New York, NT: Basic Books; 1997.

[27] McCullough L, Kuhn N, Andrews S, Kaplan A, Wolf J, Hurley CL. Treating affect phobia A manual for short-term dynamic psychotherapy. New York: Guilford Press; 2003.

[28] Monsen JT, Monsen K. Affect and Affect Consciousness: A psychotherapy Model Integrating Silvan Tomkins’s Affect–and Script Theory Within the Framework of Self Psychology. Goldberg A, editor. Pluralism in Self Psychology: Progress in Self-Psychology. 1999;15: 287–306.

[29] Jurist EL. Mentalized affectivity. Psychoanal Psychol. 2005;22: 426–444.

[30] Greenberg L. The Clinical Application of Emotion in Psychotherapy In: Lewis M, Haviland-Jones JM, Barrett LF, editors. Handbook of Emotions. The Guilford Press, New York 2008 pp. 88–101.

[31] Dimaggio G, Montano A, Popolo R, Salvatore G. Metacognitive Interpersonal Therapy for Personality Disorders: A treatment manual. Routledge; 2015.

[32] McMain S, Links PS, Guimond T, Wnuk S, Eynan R, Bergmans Y, et al An exploratory study of the relationship between changes in emotion and cognitive processes and treatment outcome in borderline personality disorder. Psychother Res 2013;23: 658–673. 10.1080/10503307.2013.838653 24156526

[33] Schanche E, Stiles TC, McCullough L, Svartberg M, Nielsen GH. The relationship between activating affects, inhibitory affects, and self-compassion in psychotherapy patients with cluster C personality disorders. Psychotherapy. 2011;48: 293–303. 10.1037/a0022012 21604900

[34] Berggraf L, Ulvenes PG, Oktedalen T, Hoffart A, Stiles T, McCullough L, et al Experience of affects predicting sense of self and others in short-term dynamic and cognitive therapy. Psychotherapy, 2014;51: 246–257. 10.1037/a0036581 24884340

[35] Solbakken OA, Hansen RS, Monsen JT. Assessment of affect integration: Validation of the affect consciousness construct. J Pers Assess. 2011;92: 257–265. 10.1080/00223891.2011.558874 21516584

[36] Mohaupt H, Holgersen H, Binder PE, Nielsen GH. Affect consciousness or mentalization? A coomparison of two concepts with reagrd to affect development and affect regulation. Scand J Psychol. 2006;47: 237–244. 16869856 10.1111/j.1467-9450.2006.00513.x

[37] Choi-Kain LW, Gunderson JG. Mentalization: Ontogeny, assessment, and application to the treatment of borderline personality disorder. Am J Psychiatry. 2008;165: 1127–1135. 10.1176/appi.ajp.2008.07081360 18676591

[38] Monsen JT, Monsen K, Solbakken OA, Sandvik-Hansen R. The affect consciousness interview (ACI) and the affect consciousness scale (ACS): Instructions for the interview and rating Oslo, Norway: Available from the Department of Psychology, University of Oslo; 2008.

[39] Monsen JT, Ødegård P, Melgård T. Major psychological changes after intensive psychotherapy: Findings from the Tøyen project, Oslo. Psychoanal Psychother 1989;7: 171–180.

[40] Monsen JT, Odland T, Faugli A, Daae E, Eilertsen DE. Personality disorders: Changes and stability after intensive psychotherapy focusing on affect consciousness. Psychother Res. 1995;5: 33–48.

[41] Monsen JT, Odland T, Faugli A, Daae E, Eilertsen DE. Personality disorders and psychosocial changes after intensive psychotherapy: A prospective follow-up study of an outpatient psychotherapy project, 5 years after end of treatment. Scand J Psychol 1995;36: 256–268. 7481598 10.1111/j.1467-9450.1995.tb00985.x

[42] Monsen K, Monsen JT. Chronic pain and psychodynamic body therapy: A controlled outcome study. Psychother Theor Res Pract Train. 2000;37: 257–269.

[43] Gude T, Monsen JT, Hoffart A. Schemas, affect consciousness, and cluster C personality pathology: A prospective one-year follow-up study of patients in a schema-focused short-term treatment program. Psychother Res. 2001;11: 85–98. 10.1080/713663854 25849879

[44] Monsen JT, Eilertsen DE, Melgård T, Ødegård P. Affects and affect consciousness: Initial experience with the assessment of affect integration. J Psychother Pract Res. 1996;5: 238–249. 22700292 PMC3330421

[45] Lech B, Andersson G, Holmqvist R. Consciousness about own and others’ affects: A study of the validity of a revised version of the affect consciousness interview. Scand J Psychol 2008;49: 515–521. 10.1111/j.1467-9450.2008.00666.x 18489533

[46] Normann-Eide E, Johansen MS, Normann-Eide T, Egeland J, Wilberg T. Is low affect consciousness related to severity of psychopathology? A cross-sectional study of patients with avoidant and borderline personality disorder. Compr Psychiatry 2013;54: 149–157. 10.1016/j.comppsych.2012.07.003 22998844

[47] Waller E, Scheidt CE. Somatoform disorders as disorders of affect regulation: A study comparing the TAS-20 with non-self-report measures of alexithymia. J Psychosom Res. 2004;57: 239–247. 15507250 10.1016/S0022-3999(03)00613-5

[48] Holmqvist R. Psychopathy and affect consciousness in young criminal offenders. J Interpers Violence 2008;23: 209–224. 18162637 10.1177/0886260507309341

[49] Lech B, Holmqvist R, Andersson G. Affect consciousness and eating disorders. Short Term Stability and Subgroup Characteristics. Eur Eat Disorders Rev 2012;20: e50–e55. 10.1002/erv.1091 21275009

[50] Lech B, Andersson G, Holmqvist R. Affect consciousness and adult attachment. Psychology 2012;3: 675–680.

[51] Solbakken OA, Hansen RS, Havik OE, Monsen JT. Affect integration as a predictor of change: Affect consciousness and treatment response in open-ended psychotherapy. Psychother Res. 2012;22: 656–672. 10.1080/10503307.2012.700871 22757634

[52] Solbakken OA, Hansen RS, Monsen JT. Affect integration and reflective function: Clarification of central conceptual issues. Psychother Res. 2011;21: 482–496. 10.1080/10503307.2011.583696 21623546

[53] Clark L. A. Stability and change in personality disorder. Curr Dir Psychol Sci. 2009;18: 27–31.

[54] Hopwood CJ, Malone JC, Ansell EB, Sanislow CA, Grilo CM, McGlashan TH, et al Personality Assessment in DSM-5: Empirical support for rating severity, style, and traits. J Pers Disord. 2011;25: 305–320. 10.1521/pedi.2011.25.3.305 21699393

[55] Kvarstein EH, Karterud S. Large variation of severity and longitudinal change of symptom distress among patients with personality disorders. Personal Ment Health. 2013;7: 265–276. 10.1002/pmh.1226 24343976

[56] Karterud S, Johansen MS, Wilberg T. Conjoint group and individual therapy in a research trial for patients with severe personality disorders. Group. 2007;31: 31–46.

[57] Arnevik E, Wilberg T, Urnes Ø, Johansen M, Monsen JT, Karterud S. Psychotherapy for personality disorders: Short-term day hospital psychotherapy versus outpatient individual therapy–a randomized controlled study. Eur Psychiatry. 2009;24: 71–78. 10.1016/j.eurpsy.2008.09.004 19097870

[58] Arnevik E, Wilberg T, Urnes Ø, Johansen M, Monsen JT, Karterud S. Psychotherapy for personality disorders: 18 months’ follow-up of the Ullevål Personality Project. J Pers Disord. 2010;24: 188–203. 10.1521/pedi.2010.24.2.188 20420475

[59] Gullestad F, Wilberg T, Klungsøyr O, Urnes Ø, Karterud S. Is treatment in a day hospital step-down program superior to outpatient individual psychotherapy for patients with personality disorders? 36 months follow-up of a randomized clinical trail comparing different treatment modalities. Psychother Res. 2012;22: 426–441. 10.1080/10503307.2012.662608 22417131

[60] Antonsen BT, Klungsøyr O, Kamps A, Hummelen B, Johansen MS, Pedersen G, et al Step-down versus outpatient psychotherapeutic treatment for personality disorders: 6-year follow-up of the Ullevål Personality Project. BMC Psychiatry. 2014;14: 119 Available: http://www.biomedcentral.com/1471-244X/14/119. 10.1186/1471-244X-14-119 24758722 PMC4000615

[61] Sheehan DV, Lecrubier Y. Mini-international neuro-psychiatry interview (M.I.N.I.) Tampa (Fla): University of South Florida Institute for Research in Psychiatry/INSERM-Hospital de la Salpétrière; 1994.

[62] First MB. Structured clinical interview for DSM-IV (version 2.0) New York: New York State Psychiatry Institute; 1994.

[63] Derogatis LR. SCL-90-R manual: administration, scoring & procedures USA: Clinical Psychometric Research; 1983.

[64] Pedersen GA. Revised Norwegian version of inventory of interpersonal problems-Circumplex (IIP-C). Tidsskr Nor Psykologforen. 2002;39: 25–34.

[65] Alden LE, Wiggins JS, Pincus AL. Construction of circumplex scales for the inventory of interpersonal problems. J Pers Assess. 1990;55: 521–536. 2280321 10.1080/00223891.1990.9674088

[66] Hudson WW. The clinical measurement package: A field manual. Chicago, IL: The Dorsey Press; 1982.

[67] Hudson WW. The Wallmyr assessment scale scoring manual. Tempe, AZ: Walmyr Publishing Co; 1992.

[68] Verheul R, Andrea H, Berghout CC, Dolan C, van Busschbach JJ, van der Kroft PJA, et al Severity indices of personality problems (SIPP- 118): Development, factor structure, reliability, and validity. Psychol Assessment. 2008;20: 23–34. 10.1037/1040-3590.20.1.23 18315396

[69] Bastiaansen L, De Fruyt F, Rossi G, Schotte C, Hofmans J. Personality disorder dysfunction versus traits: Structural and conceptual issues. Personal Disord. 2013;4: 293–303. 10.1037/per0000018 23834515

[70] Andrea H, Verheul R, Berghout CC, Dolan C, van der Kroft PJA, Bateman AW, et al. (2007). Measuring the core components of maladaptive personality: Severity indices of personality problems (SIPP-118). Report of the Viersprong Institute for Studies on Personality Disorders (VISPD) in cooperation with the Department of Medical Psychology & Psychotherapy, Erasmus University Rotterdam, The Netherlands; 2007. Available: http://hdl.handle.net/1765/10066.

[71] Feenstra DJ, Hutsebaut J, Verheul R, Busschbach JJV. Severity Indices of Personality Problems (SIPP-118) in Adolescents: Reliability and Validity. Psychol Assessment. 2011;23: 646–655. 10.1037/a0022995 21500921

[72] Arnevik E, Wilberg T, Monsen JT, Andrea H, Karterud S. A cross-national validity study of the Severity Indices of Personality Problems (SIPP-118). Personal Ment Health. 2009;3: 41–55.

[73] Cohen J. Statistical power analysis for the behavioral sciences. New York: Academic; 1988.

[74] Magai C. Affect, imagery and attachment: Working models of interpersonal affect and the socialization of emotion Cassidy J, Shaver P, editors. Handbook of Attachment. New York: Guilford Press; 1999 pp. 787–802.

[75] Conklin CZ, Bradley R, Westen D. Affect regulation in borderline personality disorder. J Nerv Ment Disord. 2006;194: 69–77. 10.1097/01.nmd.0000198138.41709.4f 16477183

[76] Gratz KL, Rosenthal MZ, Tull MT, Lejuez CW, Gunderson JG. An experimental investigation of emotion dysregulation in borderline personality disorder. J Abnorm Psychol. 2006;115: 850–855. 17100543 10.1037/0021-843X.115.4.850

[77] Feenstra DJ, Hutsebaut J, Verheul R, van Limbeek J. Identity: Empirical contribution. Changes in the identity integration of adolescents in treatment for personality disorders. J Pers Disord. 2014;28: 101–112. 10.1521/pedi.2014.28.1.101 24344891

[78] Bales D, van Beck N, Smits M, Willemsen S, van Busschbach JJ, Verheul R, et al Treatment outcome of 18-month, day hospital mentalization-based treatment (MBT) in patients with severe borderline personality disorder in the Netherlands. J Pers Disord. 2012;26: 568–582. 10.1521/pedi.2012.26.4.568 22867507

[79] Bales DL, Timman R, Andrea H, van Busschbach JJ, Verheul R, Kamphuis JH. Effectiveness of Day Hospital Mentalization-Based Treatment for Patients with Severe Borderline Personality Disorder: A Matched Control Study. Clin Psychol Psychother. 2014; 10.1002/cpp.1914 25060747

[80] Laurenceau J-P, Feldman Barrett LA, Pietromonaco PR. Intimacy as an interpersonal process: The importance of self-disclosure and perceived partner responsiveness in interpersonal exchanges. J Pers Soc Psychol. 1998;74: 1238–1251. 9599440 10.1037//0022-3514.74.5.1238

[81] Butler EA, Egloff B, Wilhelm FH, Smith NC, Erickson EA, Gross JJ. The social consequences of expressive suppression. Emotion. 2003;3: 48–67. 12899316 10.1037/1528-3542.3.1.48

[82] Rimé B. Emotion elicits the social sharing of emotion: Theory and empirical review. Emot Rev. 2009;1: 60–85.

[83] Taylor CT, Laposa JM, Alden LE. Is avoidant personality disorder more than just social avoidance? J Pers Disord. 2004;18: 571–594. 15615668 10.1521/pedi.18.6.571.54792

[84] Glenn CR, Klonsky DE. Emotion dysregulation as a core feature of borderline personality disorder. J Pers Disord. 2009;23: 20–28. 10.1521/pedi.2009.23.1.20 19267659

[85] Beblo T, Fernando S, Kamper P, Griepenstroh J, Aschenbrenner S, Pastuszak A, et al Increased attempts to suppress negative and positive amotions in Borderline Personality Disorder. Psychiat Res. 2013;210: 505–509. 10.1016/j.psychres.2013.06.036 23871409

[86] Dimaggio G, Carcione A, Nicolò G, Lysaker PH, d’Angerio S., Coni M.L., et al Differences in Axes depends on where you set the bar: associations among symptoms, interpersonal relationships and alexithymia with a number of personality disorder criteria. J Pers Disord. 2013;27: 371–382. 10.1521/pedi_2012_26_043 23130814

